# Moderately degenerated lumbar motion segments: Are they truly unstable?

**DOI:** 10.1007/s10237-016-0835-9

**Published:** 2016-09-23

**Authors:** M. M. van Rijsbergen, V. M. P. Barthelemy, A. C. T. Vrancken, S. P. M. Crijns, H.-J. Wilke, W. Wilson, B. van Rietbergen, K. Ito

**Affiliations:** 1 Orthopaedic Biomechanics, Department of Biomedical Engineering, Eindhoven University of Technology, P.O. Box 513, 5600 MB Eindhoven, The Netherlands; 2 Imaging Division, Department of Radiotherapy, University Medical Center Utrecht, Utrecht, The Netherlands; 3 Institute of Orthopaedic Research and Biomechanics, Center of Musculoskeletal Research Ulm (ZMFU), University Hospital Ulm, Ulm, Germany

**Keywords:** Spinal motion segment, Finite element analysis, Intervertebral disc, Biochemical composition, Degeneration

## Abstract

The two main load bearing tissues of the intervertebral disc are the nucleus pulposus and the annulus fibrosus. Both tissues are composed of the same basic components, but differ in their organization and relative amounts. With degeneration, the clear distinction between the two tissues disappears. The changes in biochemical content lead to changes in mechanical behaviour of the intervertebral disc. The aim of the current study was to investigate if well-documented moderate degeneration at the biochemical and fibre structure level leads to instability of the lumbar spine. By taking into account biochemical and ultrastructural changes to the extracellular matrix of degenerating discs, a set of constitutive material parameters were determined that described the individual tissue behaviour. These tissue biomechanical models were then used to simulate dynamic behaviour of the degenerated spinal motion segment, which showed instability in axial rotation, while a stabilizing effect in the other two principle bending directions. When a shear load was applied to the degenerated spinal motion segment, no sign of instability was found. This study found that reported changes to the nucleus pulposus and annulus fibrosus matrix during moderate degeneration lead to a more stable spinal motion segment and that such biomechanical considerations should be incorporated into the general pathophysiological understanding of disc degeneration and how its progress could affect low back pain and its treatments thereof.

## Introduction

The two main load bearing tissues of the intervertebral disc (IVD) are the nucleus pulposus (NP) and the annulus fibrosus (AF). Both are composed of the same major constituents, but differ in their organization and relative amounts (Antoniou et al. 1996). For healthy tissue, the NP is rich in water and proteoglycans, and less so in randomly orientated collagen network. Due to the presence of proteoglycans, a substantial osmotic pressure is sustained in the confined NP, making this tissue suitable for withstanding compressive loads (Urban and McMullan 1985). The AF contains much collagen fibres, which are highly aligned in lamellae, having a preferred direction of ±30${^{\circ }}$ with respect to the endplate. It has some proteoglycans. Due to the anisotropic structure of the fibres in the AF, together with the swelling capacity of the NP, the IVD is able to withstand complex loading conditions (Meakin and Hukins 2000).

With degeneration, the clear distinction between NP and AF disappears, leading to a more fibrotic IVD, which is less able to withstand these complex loading conditions. In the earliest stages of degeneration, the NP starts to lose proteoglycans (Antoniou et al. 1996). Due to this loss, the NP swelling capacity decreases significantly and permeability of the NP tissue increases. These changes also result in a drop in compressive stiffness of the NP (Johannessen and Elliott 2005). Hence, the NP is not able to withstanding high loads for a prolonged period of time. Simultaneously, the organization of the AF becomes less structured, that is fewer fibres are orientated in the preferred collagen orientation (Gu et al. 1999; Iatridis et al. 1998; Dittmar et al. 2016), and due to the loss of osmotic pressure in the NP, the inner AF bends inward.

The changes in biochemical content lead to changes in mechanical behaviour of both tissues (Fujita et al. 1997; Johannessen and Elliott 2005; O’Connell et al. 2012), influencing the overall behaviour of the intervertebral disc and the corresponding spinal motion segment (SMS). With progressive degeneration, other morphological changes occur such as the formation of osteophytes, severe height loss of the IVD and endplate sclerosis (Wilke et al. 2006), which can also influence the behaviour of the SMS.

For severe degeneration, independent of the different classification systems, these changes lead to stiffening of the SMS compared to a healthy motion segment, as first described by Kirkaldy-Willis and Farfan (1982) and likewise found by others (Kettler et al. 2011; Kettler and Wilke 2004; Kirkaldy-Willis and Farfan 1982; Mimura et al. 1994; Oxland et al. 1996; Tanaka et al. 2001). Where stability/instability in these studies is defined according to Pope and Panjabi (1985) as an abnormal response to physiological loads, with reduced spinal stiffness and greater motion compared to the healthy SMS. In contrast, for early degeneration, that is the first stages in which the degeneration starts to become clearly visible (for example Pfirrmann grade III), contradictory observations are described in the literature (derived during in vitro experiments). Some studies have reported instability for mild degeneration (Fujiwara et al. 2000; Krismer et al. 2000; Tanaka et al. 2001), while other studies have showed increasing spinal stiffness with progressing degeneration (Kettler et al. 2011; Oxland et al. 1996). Although not all studies were conducted using the same loading configuration and set-up, the trend in outcome is sometimes in contrast to each other. This contradiction is also found in finite element (FE) studies (Galbusera et al. 2011a, b; Rohlmann et al. 2006), which investigated the effect of different morphological changes during degeneration. These FE studies were based on models which take into account general phenomenological changes in mechanical properties as described in the literature and assume a percentage in height reduction of the IVD based on general classification schemes. However, they do not take into account the more well-characterized changes in biochemical content and collagen anisotropy. Furthermore, in all cases, the kinematics were investigated only in the three main principal bending directions. In daily life, however, shear loads play a major role in the loading configuration of the lumbar spine (Potvin et al. 1991) and can have contribution to spinal instability (Melnyk et al. 2015).

Recently, we showed that our fibre-reinforced osmoporo-viscoelastic (FR-OPVE) multi-scale cartilaginous tissue model, based on matrix constituent contents and their material properties, could be used to simulate the kinematic behaviour of a healthy SMS (Barthelemy et al. 2016). The aim of the current study was to use this model to simulate the tissue behaviour of a moderately degenerated SMS and investigate if moderate degeneration at the biochemical and fibre structure level leads to instability, that is an abnormal response to physiological loads, with reduced spinal stiffness and greater motion compared to the healthy SMS (Pope and Panjabi 1985), of the lumbar spine. We hope to contribute to the current understanding of and discussion related to moderate disc degeneration, its role in spinal stability and the implications and treatments thereof.

## Material and method

First, individual tissues of the healthy SMS (hSMS) model (Barthelemy et al. 2016) were adapted to a moderately degenerated disc by taking into account the change in constituent content of the NP and AF tissue. The constitutive material parameters for degenerated tissues were updated by fitting the model to experimental data of the individual tissues from moderately degenerated discs and accordingly implemented together with biochemical content changes into the hSMS model. Second, the kinematics of this degenerated SMS (degenSMS) model was compared to the kinematic behaviour of (1) in vitro studies reported in the literature and (2) against our own in vitro results. Finally, based on the difference in kinematic behaviour between the healthy and degenerated SMS, the main question of this study was answered.

### Determination of unknown constitutive material parameters

#### Human moderately degenerated lumbar disc material testing

Four fresh frozen, human cadaveric lumbar spines (all male, mean age 81 years; Table 1) were obtained from the Department of Anatomy, University Medical Center Utrecht, the Netherlands, according to institutional guidelines. After thawing, the specimens were imaged with a 1.5 Tesla whole body MR system combined with a Synergy body coil (Achieva, Philips Medical Systems, Best, the Netherlands). T2-weighted turbo spin echo sagittal images, without fat suppression, were acquired (TR $=$ 3500 ms, TE $=$ 120 ms) according to the protocol by Griffith et al. (2007): matrix $=$ 512 $\times$ 281, field of view $=$ 320 mm $\times$ 320 mm, slice thickness $=$ 4 mm, inter-slice gap $=$ 0.4 mm, number of scan averages $=$ 4, turbo spin echo factor $=$ 17. The spines were stored at $-25 {^{\circ }}\hbox {C}$ until tissue dissection. From the MR images, a senior orthopaedic spine surgeon assessed the degree of IVD degeneration, according to the modified Pfirrmann grading scale (Table 1). Discs suffering from grade III, IV and V degeneration were considered moderately degenerated and prepared for mechanical testing. After overnight thawing, soft tissues, tendons and ligaments were removed and the IVD was released from the vertebrae. To prevent any swelling, harvested discs were immediately sealed in a double plastic layer and stored at $-30\,{^{\circ }}\hbox {C}$ until further use.

**Table 1:** Modified Pfirrmann grades for the five lumbar IVDs in each spinal segment. The IVDs selected for mechanical testing are printed in boldface

Spinal level	Age, gender			
	87	77	72	87
L1–L2	VII	**V**	**IV**	**III**
L2–L3	VIII	**V**	II	**IV**
L3–L4	VII	**V**	**III**	**V**
L4–L5	**IV**	**III**	**V**	VI
L5–S1	VII	II	II	**IV**

**Fig. 1:**
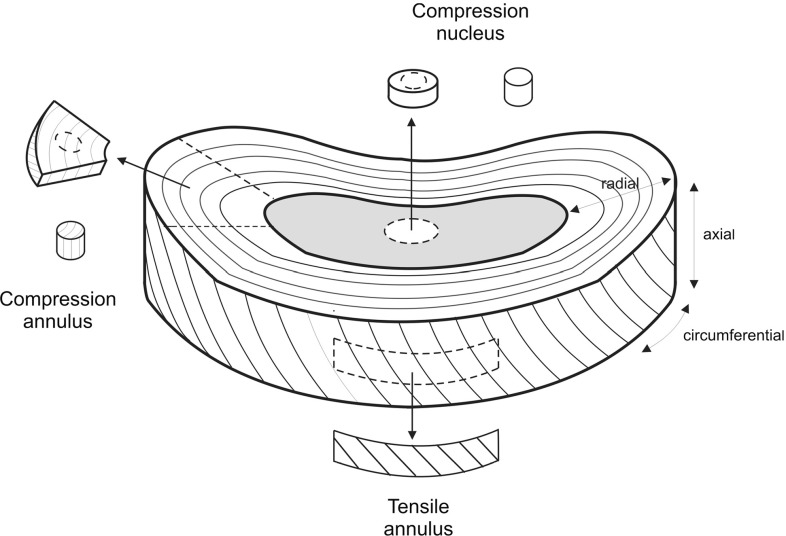
Orientation and location of the test samples

Mechanical testing was conducted on individual NP and AF tissue specimens. Two types of mechanical tests were performed: (1) confined compression (CC, with NP and AF tissue) and (2) uniaxial tension (AF only). For CC, samples with axial orientation were prepared from the central region of the NP and the lateral region of the AF. For tensile tests, circumferentially oriented samples were prepared from the central anterior region of the AF (Fig. 1).

#### Confined compression experiments

While kept frozen, samples were cut to size (Ø 5 mm, height 1.5 mm) and inserted in a custom-built CC setup, based on the design of Best et al. (1994). The confining chamber was filled with 0.15 M phosphate buffered saline (PBS). After isometric thawing for 5 min, the sample was compressed to a 3 % strain preload (at 0.25  $\upmu \hbox {m}/\hbox {s}$), using a porous filter (Duran P4, Schott, Mainz, Germany, pore size 10–16 $\upmu \hbox {m}$, permeability $1.15\times 10^{-7}$ $\hbox {m}^{4}/\hbox {Ns}$) connected to a materials testing system (Z010, Zwick Roell, Ulm, Germany) via a hollow piston, and allowed to reach equilibrium for 4 h. Thereafter, an additional 5 % strain was applied (at 0.25 $\upmu \hbox {m}/\hbox {s}$) and stress relaxation was recorded for 4 h. Only the stress relaxation data was processed further. The maximum strain value was chosen in line with other studies (Johannessen and Elliott 2005) and was assumed to be realistic under daily normal loading conditions, for example standing.

#### Uniaxial tensile experiments

Using a custom cutting tool, AF specimens of $15 \times 2 \times 1.5$ mm ($l \times w \times h$) were prepared. Sandpaper grips were glued to the samples’ ends to prevent slippage when being loaded. The specimens were mounted to a materials testing system (Z010, Zwick Roell, Ulm, Germany) using clamps connected to the system’s crosshead and base plate. To prevent dehydration of the sample, the specimen was surrounded by a custom designed testing chamber filled with 0.15 M PBS. Following 15 min creep at a tare load of 0.1 N, specimens were subjected to 12 preconditioning cycles between either 0–6 % or 0–10 % strain (at 0.01 $\hbox {s}^{-1}$). Each preconditioning protocol was followed by 15 min equilibration at 0 % strain to return to the 0.1 N tare load. The final experiment consisted of two stress relaxation tests, the first up to 6 % strain and the second up to 10 % strain (at 0.15 mm/s), both allowing 15 min relaxation. The two strain values were chosen such that a clear difference in tensile behaviour between the two experiments could be found.

#### Finite element simulations

To simulate experiments, simplified FE meshes were used to reduce the computation time, similar to Barthelemy et al. (2016). Due to initial swelling, fibres are put under tension and consequently restricting the swelling of the tissue (Wilson et al. 2007). During the compression phase of the confined compression experiments, the fibres are within the unloading regime of the tensile curve and therefore contribute to the compression stiffness of the tissue, i.e. the fibres are still under tension during compression. To account for the increase in collagen content due to degeneration, the additional collagen content compared to the healthy tissue (Barthelemy et al. 2016) was assumed to be strain free during the swelling process, that is no pre-tensioning due to swelling is occurring in these fibres. As such, stiffening of the tissue during residual swelling was prevented, mimicking the natural degeneration process. All experimental steps were simulated by applying the above-mentioned loading velocities and equilibration times. Reaction forces, for AF CC and AF tensile tests, and subsequent stress relaxations, for NP CC, were fitted to the corresponding experimental averages, similarly as in Barthelemy et al. (2016).

#### Fitting procedure

Constituent material parameters values of the individual healthy intervertebral disc tissue were used as the starting points (Barthelemy et al. 2016). First, similar to Barthelemy et al. (2016) the NP confined compression experiment was simulated. In this step, the permeability constants as well as the constituent shear modulus of the non-fibrillar matrix were determined. The initial values were adjusted till the results of the numerical simulation, that is reaction stress, fell within one standard deviation of those of the experiments using an unconstrained nonlinear optimization procedure in Matlab, similar to Schroeder et al. (Schroeder et al., 2007). Second, based on this outcome the parameters were manually fine-tuned to get a best match between experimental and numerical behaviour. After determination of the shear modulus of the non-fibrillar matrix and the permeability constants, these parameters were fixed and the other six constituent parameters were determined using the experimental data from the AF samples. Again, the same optimization steps were followed. The obtained constituent material parameters were compared to the set obtained for healthy tissue.

The constitutive laws of the FR-OPVE model directly incorporate parameters describing the collagen content, FCD and fluid fraction (Wilson et al. 2006). For details, see (Barthelemy et al. 2016; Wilson et al. 2007). Similar to Barthelemy et al. (2016), the structure of the collagen fibre network in the AF was assumed to be composed of two main primary fibre directions, oriented within the lamellae planes, and thirteen secondary fibre directions corresponding to an isotropically oriented smaller fibre structure. The two main primary fibres were assumed to be similar to the healthy structure ($\pm 23{^{\circ }}$ in circumferential direction at anterior location (Dittmar et al. 2016). The relative collagen fraction for primary and isotropically orientated fibre directions were given by (Eq. 1)

1 $$\begin{aligned} \rho _{c,1}= & {} \rho _c \frac{C}{2C+13} \text { for primary fibre directions}\\ \rho _{c,2}= & {} \rho _c \frac{1}{2C+13} \text { for isotropically oriented fibre directions}\nonumber \end{aligned}$$

For primary fibres, $\rho _c$ equals 2, for isotropically oriented fibre directions, $\rho _c$ equals 13.The ratio between primary and isotropically orientated fibre directions was characterized with *C*. For healthy AF tissue, this parameter was estimated to be 38 (Barthelemy et al. 2016; Schroeder et al. 2010), indicating a ratio of 85:15 between primary and isotropically orientated fibre directions. For degenerated tissue, the ratio between primary and isotropically oriented fibre directions in the AF is 70:30, that is $C = 15$ (Dittmar et al. 2016). The collagen fibres in the NP were assumed to be organized isotropically oriented (for both healthy and degenerated tissue). The isotropically orientated fibre structure was assumed to be homogeneous and was represented by 13 fibre directions, 3 along the *x*-, *y*- and *z*-axis directions, 6 in all directions with $45{^{\circ }}$ angle to the *x*-, *y*- and *z*-axis (Wilson et al. 2004) within the *xy*, *xz* and *yz* planes and 4 at equal angles between the *x*-, *y*- and *z*-axes.

By only taking into account biochemical and structure changes (C and collagen fibre angle), no acceptable fit between the experimental and FE reaction forces/stresses could be obtained (data not shown). Thus, new constituent material parameters for degenerated tissues were fitted. The constituent properties, which describe the non-fibrillar ($$G_{mf} $$, $$G_{mnf} $$, *M* and $$\alpha $$) and the collagen fibre ($$E_1 $$, $$E_2 $$, $$k_1 $$, $$k_2 $$ and $$\eta $$) properties (Barthelemy et al. 2016; Wilson et al. 2006), were determined such that the four described mechanical tests were simulated within the standard deviation of the experiments. The biochemical content and structure changes for Thompson grade III discs (which corresponds to Pfirrmann grade III, IV, V (Griffith et al. 2007)) for the individual tissues were taken from the literature (Antoniou et al. 1996, 2004; Johannessen and Elliott 2005; Lyons et al. 1981; Dittmar et al. 2016) (Table 2).

**Table 2:** Biochemical and compositional composition of generic grade III IVD

	Nucleus pulposus	Annulus fibrosus
Water (% ww)	76	69
Collagen (%dw)	30	78
FCD (mEq/ml)	23	19
Proportionality (C)	–	70:30
Collagen fibre angle (${^{\circ }}$)	–	23

**Table 3:** Constitutive material parameters for a healthy IVD and a grade III IVD (Pfirrmann et al. 2001)

	Fibre properties						Ground substance			
	*C* (–)	$E_{1}$ (MPa)	${k}_{1}$ (–)	$E_{2}$ (MPa)	${k}_{2}$ (–)	$\eta$ (MPa s)	${G}_{{mnf}}$ (MPa)	${G}_{{mf}}$ (MPa)	${M}_{{k}}\,$(–)	$\alpha$ ($\hbox {mm}^{4}/\hbox {Ns}$)
Healthy IVD	38	1.62	9.88	1.44	8.2	780	1	1	0.9	1.3e−4
Degen IVD	15	3.6	11	1.7	8.8	1600	0.9	1	1.2	2.5e−4
% Difference		$+$125	$+$11	$+$25	$+$7	$+$100	−10	–	$+$25	$+$92

### Spinal motion segment kinematics

#### Comparison against literature

The SMS as described by Barthelemy et al. (2016) was used as starting point to evaluate the behaviour of a degenSMS. First, disc height loss was simulated after considering changes in composition and constitutive properties of the degenerated disc under a compressive load of 250 N for 1 hour. If this height loss was less than that measured by Murata et al. (1994), the disc height was manually reduced (by adjusting the pre-swollen mesh) till a height loss of 8–10 % was obtained.

The mechanical behaviour of the degenerated SMS was investigated under pure moments of 7.5 Nm in the three main bending directions. Simulated range of motion (RoM), neutral zone (NZ) and stiffness (Wilke et al. 1998) were compared to the behaviour of the healthy SMS, in vitro studies (Kettler et al. 2011; Oxland et al. 1996) and against our own experimental data of three mildly degenerated monosegments L3–L4 (see below). Outcome was evaluated in terms of relative behaviour (difference between healthy and degenSMS) as well as for absolute values.

In addition to these loading directions, an anterior-to-posterior shear load of 250 N with an axial compressive force of 300 N (Melnyk et al. 2015) was applied to both the healthy and degenerated SMS. Displacement of the L3 vertebra, relative to L4, was evaluated for both situations. The healthy SMS was compared to the literature (Melnyk et al. 2015) and was used as reference value. Next, also the difference in foramen dimension (Hasegawa et al. 1995) was investigated between hSMS and degenSMS.

#### Comparison against in vitro experiments

In addition to the literature data, experiments were performed to determine the kinematics of three moderately degenerated human lumbar monosegments L3–L4. These specimens were obtained from the Department of Anatomy, University Medical Center Utrecht, the Netherlands, according to institutional guidelines. All of them were male, with a mean age of 61 years (range 52 – 66). Postmortem, lumbar spines were stored at $-25\,{^{\circ }}\hbox {C}$ until the moment of use. L3–L4 specimens were harvested including all ligaments and para-spinal muscles. Discs were classified as grade 2 and 3 according to the Pfirrmann grading scheme (Pfirrmann et al. 2001).

Experiments were done similarly as described by Wilke et al. (1998). Specimens were affixed to a custom-built spine tester (Wilke et al. 1994) and loaded with pure unconstrained moments of ±7.5 Nm to move towards flexion and extension assessing the absolute neutral position. Next, a compressive preload of 250 N was applied for one hour to simulate physiological conditions in the upright standing posture and to minimize the effects of a superhydrated intervertebral disc (Wilke et al. 1998). After one hour, while keeping the compressive load in place, pure unconstraint moments of 7.5 Nm were applied in three principle directions (flexion/extension, lateral bending and axial rotation). In each direction, two full cycles were completed for preconditioning and data of the third cycle was used for analysis. Moments and angles of both vertebrae were recorded using a 3D Vicon motion-tracking system. From this data, RoM and NZ were calculated to which the behaviour of the degenSMS was compared (Wilke et al. 1994, 1998).

## Results

### Determination of unknown constitutive material parameters

**Fig. 2:**
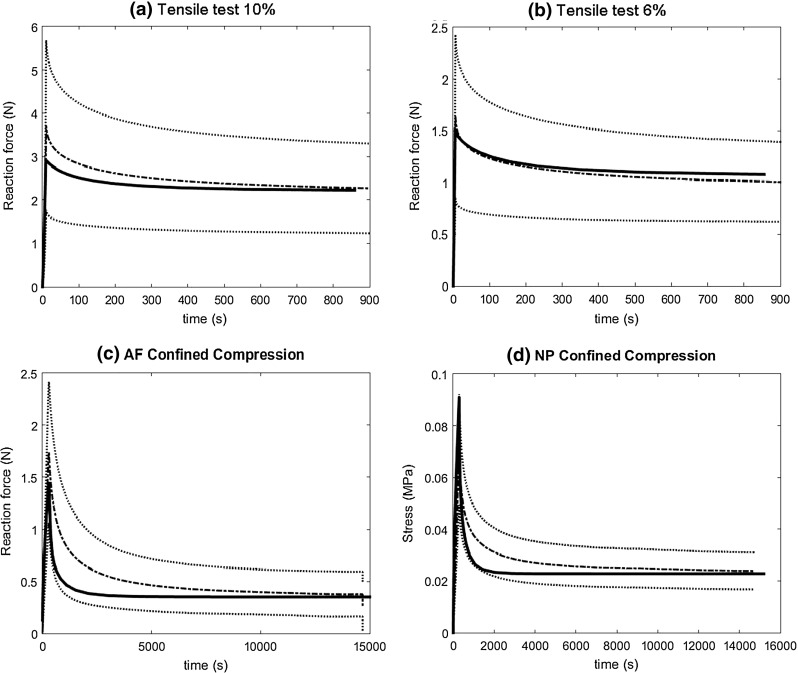
Comparison of the fitted reaction forces/stresses to the experimental data for tensile test of annulus fibrosus (AF) tissue at 10 % (**a**) and 6 % strain (**b**) and confined compression tests of annulus tissue (**c**) and nucleus pulposus (NP) tissue (**d**). The *dashed lines* represent the experimental data, where the *dashed-continuous line* represents the mean value and the *dashed line* the standard deviation. The *thick continuous line* represents the numerical simulation

By assuming moderately degenerative structure and biochemical changes, the calculated set of constitutive material parameter values were close to the set of healthy parameter values, although there were some differences (Table 3). Both tensile (Fig. 2a, b) and confined compression test simulations (Fig. 2c, d) were in good agreement with the experimental stress- and force-relaxation curves. Only the relaxation time of both NP and AF confined compression simulations was faster than experimentally measured (Fig. 2c, d). Compared to healthy disc tissue, the most relevant change in parameter value was the significant increase of the fibre stiffness $(\hbox {E}_{1})$ by 125 %. The viscoelastic behaviour of the collagen, stiffness of the non-fibrillar matrix and ground substance permeability ($\eta , \hbox {M}_{\mathrm{k}}$ and $\alpha$, respectively) also increased (Table 3).

### Spinal motion segment kinematics

#### Literature

By taking into account the content, structure and constitutive property changes, a height loss of 9 % was found when PG swelling was balanced by collagen tension. Thus, by only changing the biochemistry, collagen composition and corresponding constituent material parameters, realistic height changes were obtained.

**Table 4:** Model prediction for range of motion (RoM) for the three principle bending directions compared to in vitro experiments (Kettler et al. 2011) of comparable degeneration grade: ‘*Literature RoM*’. Numerical difference of the degenSMS model compared to hSMS model (Barthelemy et al. 2016) *‘degenSMS – hSMS Model difference*’ as well as literature data (Kettler et al. 2011) ‘*Literature difference’*. A positive difference indicates a larger RoM for the degenSMS

	degenSMS Model RoM	Literature RoM	degenSMS – hSMS Model difference	Literature difference
Flexion/extension	$4.1{^{\circ }}$	$8.5{^{\circ }}\pm 4.5{^{\circ }}$	$-0.2{^{\circ }}$	$-1{^{\circ }}\pm 3.5{^{\circ }}$
Axial rotation	$2.4{^{\circ }}$	$2.1{^{\circ }}\pm 1{^{\circ }}$	$+0.5{^{\circ }}$	$+0.2{^{\circ }}\pm 2.7{^{\circ }}$
Lateral bending	$2.7{^{\circ }}$	$4{^{\circ }}\pm 1.8{^{\circ }}$	$-0.8{^{\circ }}$	$-0.7{^{\circ }}\pm 3.2{^{\circ }}$

The RoM and stiffness of the degenSMS in flexion/extension were similar to that of a healthy SMS. For axial rotation, the degenSMS was less stiff compared to the healthy SMS (Table 4, 5), leading to a larger RoM. While for lateral bending, the stiffness increased leading to a lower RoM. The NZ of the degenSMS was comparable to the healthy SMS in all three directions and fell within the NZ values reported in literature. The relative difference between a healthy and moderately degenerated SMS was in line with the literature for all three principal bending directions (Table 4).

**Table 5:** NZ of degenSMS versus literature studies and stiffness of degenSMS versus hSMS

	Flexion/extension	Axial rotation	Lateral bending
NZ (${^\circ }$)			
Simulation	1.4	0.4	0.9
Kettler et al. (2011)	1.8 ± 1	0.2 ± 0.5	1.8 ± 1
Oxland et al. (1996)	1.4 ± 0.5	0.2 ± 0.1	4.6 ± 0.8
Stiffness (healthy/degen) (Nm/degree)			
Simulation	5.5/5.4	4.8/4.1	3.5/3.8

#### Model response to shear load

The displacement of the healthy SMS under 250 N shear load in anterior direction was comparable to the results of (Melnyk et al. 2015) (0.88 resp. 0.6±1 mm). When the same shear load was applied to the degenSMS, a displacement of vertebra L3 of 0.96 mm in the same direction was measured. Due to this displacement, the superior foraminal width (Hasegawa et al. 1995) was reduced by less than 1 % in the degenSMS compared to the hSMS.

#### In vitro experiments

**Fig. 3:**
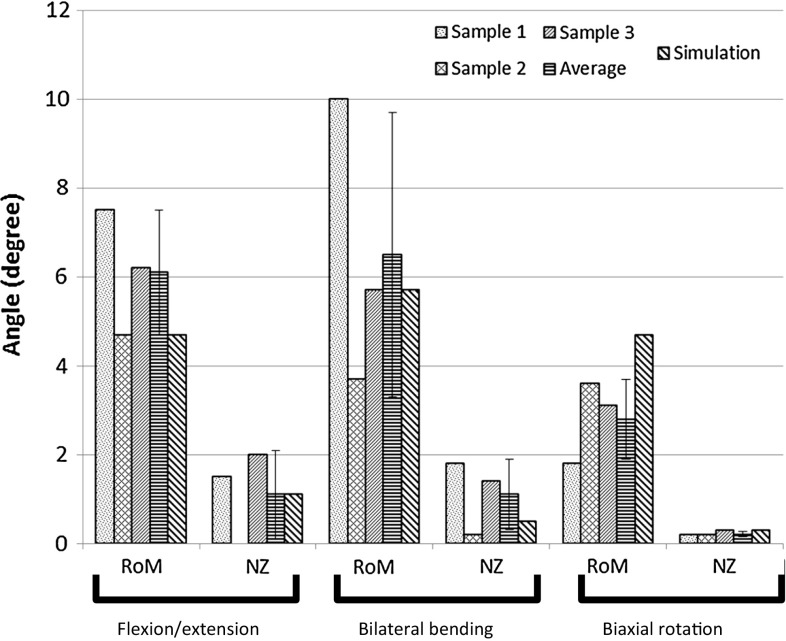
Outcome of the in vitro test in the three principle bending directions for the three specimens and the average of these experiments with corresponding standard deviation. Note that the results are for two-sided bending and rotation. The outcome of the generic degenSMS is also included in this figure

The kinematic behaviour of the three cadaveric motion segments was comparable to each other in flexion/extension and lateral bending. For bilateral bending, the outcome of the three samples was more diffuse. The generic degenSMS captured the experimental behaviour well in RoM and NZ in bilateral bending and flexion/extension. In biaxial rotation, the RoM of the model exceeded that of the experiments, although the NZ fell within the experimental range (Fig. 3).

## Discussion

Uniaxial tensile tests together with confined compression test were performed on NP and AF tissue of moderately degenerated IVDs to determine the unknown constitutive material parameters describing these tissues. Based on biochemistry and structure changes from literature, a reasonable set of constituent material parameter values was obtained. By taking into account the individual tissue changes for the moderately degenerated IVD, that is biochemistry, constituent material parameters and proportionality parameters, a realistic geometry of a degenSMS was obtained. With moderate IVD degeneration, an increase in RoM, that is instability, was found in axial rotation. However, in the other bending and movement (shear) directions a stabilizing or no significant effect of IVD degeneration was observed.

Similar to that found by Barthelemy et al. (2016), different sets of parameter values could be obtained to fit the tensile and confined compression experiments equally well, that is the set of parameters was not unique. By changing the collagen architecture from an anisotropic to a more isotropic orientation (Gu et al. 1999; Iatridis et al. 1998; Dittmar et al. 2016), a better fit was found where the constituent parameter values were least different to those of the healthy tissue (Fig. 2). Nevertheless, fibre parameters $\hbox {E}_{1}$ and $\eta$ increased significantly in comparison with the healthy set of constituents (Table 3). As mentioned in the study of Barthelemy et al. (2016), it is difficult to judge each individual parameter on its contribution to the tissue behaviour; as a set, the constituent material parameters give reasonable tissue behaviour. However, based on the known changes due to degeneration, it is expected that the stiffness of the collagen fibres would increase due to an increase in the amount of crosslinking causing a reduction in flexibility of the fibres (Duance et al. 1998), as well as an increase of collagen type I (Antoniou et al. 1996) which is stiffer than collagen type II. When these observations are translated to the constituent material model, an increase in linear stiffness ($\hbox {E}_{1})$ and strain dependent behaviour ($\hbox {k}_{1})$ is expected, as there is no specific parameter related to the amount of crosslinking or a different collagen type. Due to the change in the amount of crosslinking and collagen type, also the material constants that describe the viscoelastic behaviour of the collagen fibres are expected to change ($\hbox {E}_{2}$, $\hbox {k}_{2}$, $\eta$) as could be observed by a small, non-significant percentage change ($\hbox {E}_{2}$, $\hbox {k}_{2})$ compared to the healthy constituent material parameter values (Barthelemy et al. 2016). The viscoelastic parameter $\eta$ changed significantly by 100 % compared to the healthy constituents; however, this change is not as relevant as change of the other parameters, as the influence of this parameter on the individual tissue behaviour is small (Barthelemy et al. 2016).

The ground substance decreased in stiffness and became more permeable with degeneration, which was anticipated. As the disc degenerates, one of the earliest changes to occur is the breakdown of aggrecan by proteolytic enzymes such as ADAMS-TS4 and ADAMS-TS5 (Sztrolovics et al. 1997). These processes convert the ground substance from large molecular PG-hyaluronan aggregates to smaller partial aggrecan monomers consisting of GAG complexes attached to the GAG domain of the aggrecan protein. With this drop in molecular weight, the polymeric network of the ground substance could be expected to become less stiff and more permeable.

To determine the set of constitutive material parameter values, two types of experiments were conducted: confined compression and uniaxial tensile tests. One could argue about the correctness of this choice, that is how realistic are these experiments to mimic the natural setting of AF and NP tissues inside the IVD. However, to determine the constituent material parameter values, it is not of utmost importance how well the conducted experiments mimic the natural situation but more relevant how well they are suited to determine a particular parameter value. For example, by conducting a confined compression experiment, the parameters that could be determined are the constituent material ground substance stiffness and the permeability parameters (${G}_{\mathrm{m}}$, ${M}_{\mathrm{k}}$ and $\alpha )$. The same argumentation holds for the choice of the uniaxial tensile experiments. They are well suited to determine the collagen parameters.

For the tensile tests conducted on the AF tissue, isolated tissue samples were used (no connection of collagen fibres to the endplates). These samples were not used to determine the mechanical behaviour of the tissue itself as was done in the study of Holzapfel et al. (2005), but they were used as comparison to computational models in which the tensile tests were simulated. Based on this comparison, the mechanical properties of the tissue constituents were calculated so that the models could correctly simulate the tensile test. This approach, similar to that of Barthelemy et al. (2016) results in a mechanical tissue behaviour that depends on the constituent mechanical properties, constituent content and structure. As was shown by Barthelemy et al. (2016) for healthy disc tissue, this approach results in well corresponding disc and spinal motion segment behaviour compared to literature.

The uniqueness of the determined set of constituent material parameter values is not guaranteed (see also Barthelemy et al. 2016). Nevertheless, as the goal was not to determine their unique values but to find a collection of these parameter values that would provide good results for mechanical behaviour at the tissue level, this lack of uniqueness is justified. Finding unique values for these individual parameters would require data from more diverse (for example dynamic) experimental tests at the tissue level. Only if such data is available, would it be worthwhile to improve the fitting procedure, to test for the uniqueness of the solution, and to determine the error in these parameter values.

The individual constituent material parameters are difficult to compare to other conducted studies. The reason for this is the use of different models (for example Malandrino et al. 2015). Although similar parameter names may be used, all other models refer to tissue properties where as in this model, the parameter values are that of the tissue constituents and not of the tissue. Thus, to make comparison, one would also need to know the content of all constituents in the tissue as well as the structure of the fibre network.

In this study, the anisotropic architecture of collagen was changed, by reducing the proportion between primary and isotropically oriented fibres from 85:15 to 70:30. This was well in line with the study of Dittmar et al. (2016) where the change in collagen fibre architecture due to degeneration was studied. Also in that study, a small change in collagen fibre orientation was observed ($21{^{\circ }}$ vs. $23{^{\circ }}$ for degenerated vs. healthy AF tissue). As the standard deviation for the collagen fibre angle was large $(8.2{^{\circ }})$, it was justified to not change the fibre orientation in the degenerated model.

By taking into account the individual tissue changes, i.e. biochemistry, constituent material parameters and proportionality parameters, a disc height loss of 9 % occurred in the non-loaded equilibrium state similar to a 8–10 % disc height loss measured by Murata et al. (1994) in patients. This indicates that the model could mimic the clinically observed mass loss by taking into account the most significant tissue changes.

With moderate degeneration, a small change in RoM was observed between healthy and degenerated SMS for flexion/extension. For axial rotation and lateral bending, the degenSMS showed a weaker or respectively stiffer behaviour compared to the healthy SMS. The kinematics of this degenSMS fell within the experimental data (Tables 4, 5) as reported in literature (Kettler et al. 2011; Oxland et al. 1996) and a similar trend as reported by others, for example (Muriuki et al. 2016). In flexion/extension the behaviour was slightly too stiff (Tables 4, 5), compared to the behaviour of the healthy SMS (Barthelemy et al. 2016). For the other two bending directions, the behaviour was in line with these studies. However, there is an uncertainty about these in vitro derived data. Some studies (Kettler et al. 2011; Mimura et al. 1994; Oxland et al. 1996) showed a decrease in flexibility, already with early degeneration, which is in contradiction with the theory of Kirkaldy-Willis and Farfan (1982). However, others (Kong et al. 2009; Tanaka et al. 2001) showed that the theory of Kirkaldy-Willis and Farfan (1982) could actually be corroborated during in vitro tests. Therefore, care should be taken with the interpretation of the comparison of the results between the literature and the model as literature does not provide an unequivocal answer to the question whether early IVD degeneration causes spinal instability

No sign of instability was found in lateral bending, flexion-extension and in shear load. These observations were also found by Muriuki et al. (2016) in which no sign of instability was found for these two bending directions. Similar to results of this study, an increase in RoM for axial rotation was found. A possible explanation why no sign of instability could be expected in lateral bending and flexion-extension could be the reduction of disc height compared to the healthy state. Due to the disc height loss, the facet joints are closer to each other, limiting the flexibility in these directions and thus resulting in stiffening. This reduction of disc height could also lead to overloading of cartilage at the facet joints, resulting in its degeneration, similar to that observed by Fujiwara et al. (2000). However, it could also be argued that degeneration of cartilage could lead to a change in loading configuration at the motion segment, resulting in a stiffening or instability.

The results of the generic degenSMS model matched the outcome of the in vitro experiments reasonably well (Fig. 3, simulated value within the standard deviation of the experimental results), although the behaviour of the model in axial rotation was slightly too compliant, leading to an overestimation of the RoM (overestimation of 50 % compared to mean experimental value). A possible explanation for this discrepancy could be the different lumbar levels (Muriuki et al. 2016) that were tested as well as the small number of tested specimens. As the SMS models, both healthy as degenerated, are sensitive to geometrical differences, this could explain the difference between model and experiment.

In addition to applying pure moments in the three main bending directions, also an anterior-to-posterior shear force was applied. In the latter case, we observed a small increase in displacement of the upper vertebra for the degenerated compared to the healthy SMS. This is probably due to the change in anisotropy which is taken into account for the degenSMS. Because of this, there is a more isotropic orientation of the fibre directions, making it easier to move in the direction of the applied load compared to the healthy SMS, similar as could be observed for axial rotation. The outcome of the healthy SMS expressed in displacement of the vertebra L3 was comparable to the study of Melnyk et al. (2015), although the mean value was overestimated by 50 %. Such discrepancies might arise from the geometry differences between the spinal segment of the numerical model (L3–L4) and the experiments (L2–L5) or the limited number of specimens tested. Although we assume that our model is capable of predicting realistic displacement curves under shear load, there is no clinical relevance of the increased displacement as the dimensions of the intervertebral foramen hardly change. As the nerve takes approximate 30 % of the space of the foramen (Hasegawa et al. 1995), this observed decrease will not lead to compression of nerves that could lead to pain and therefore has no clinical relevance.

There are some limitations of this study which needs to be taken into account. First, similar to the healthy SMS, no radial gradient in AF fibre orientation was assumed, that is no change in fibre orientation was implemented going from the outer AF towards the NP, nor a distinction between inner and outer annulus. However, it is known that there is a smooth radial gradient in fibre orientation. As different studies showed that this gradient has an influence on the kinematic behaviour of the SMS (Malandrino et al. 2013; Noailly et al. 2011; Schmidt et al. 2005), this should be implemented in the future. However, although it is observed that fibre orientation in the AF locally changes with degeneration (Guerin and Elliott 2006; Dittmar et al. 2016), large standard deviations on the obtained results blurred the results found in these studies, leading to large uncertainties in the amount of changes in fibre orientation and fibre gradient. Therefore, no change in gradient was implemented compared to the healthy SMS (Barthelemy et al. 2016). Next to that, no changes in the bony endplate (Benneker et al. 2005; Roberts et al. 1996) or changes on the facet joints (Fujiwara et al. 2000), which occur during degeneration, were included, nor did we include the formation of osteophytes (Galbusera et al. 2011b). All these changes can have an influence on the kinematics of the motion segment. Second, our own in vitro study consisted only of three specimens. From a statistical point of view, no conclusion can be drawn from such a small population. However, we used this data in addition to that from the literature as there are only a small number of studies available and did not draw any conclusion from this data alone. Nevertheless, the behaviour of these three samples was in line with the most comprehensive study, which consisted of a retrospective analysis of a large database of in vitro results (203 motion segments), which showed no instability with moderate degeneration (Kettler et al. 2011).

In conclusion, we were able to capture the individual mechanical behaviour of moderately degenerated NP and AF tissue and derive their constitutive material parameter values by considering the most important tissue matrix structural and architectural changes occurring during early IVD degeneration in our FR-OPVE multi-scale cartilaginous tissue model. Incorporation of the degenerated IVD tissue models into a degenSMS model resulted in simulations of the kinematic behaviour in line with the literature results of basic bending experiments. Instability of the degenerated SMS was only found in axial rotation. Stiffening or no effect of disc degeneration was found in the other principal bending directions. When a shear load was applied to the degenerated SMS, also no sign of instability was found. This study found that reported changes to the NP and AF matrix during moderate degeneration lead to a more stable spinal motion segment, and that such biomechanical considerations should be incorporated into the general pathophysiological understanding of disc degeneration and how its progress could affect low back pain and its treatments thereof.
